# Silicon Promotes Exodermal Casparian Band Formation in Si-Accumulating and Si-Excluding Species by Forming Phenol Complexes

**DOI:** 10.1371/journal.pone.0138555

**Published:** 2015-09-18

**Authors:** Alexander T. Fleck, Sascha Schulze, Martin Hinrichs, André Specht, Friedrich Waßmann, Lukas Schreiber, Manfred K. Schenk

**Affiliations:** 1 Institute of Plant Nutrition, Faculty of Natural Sciences, Leibniz University Hannover, Herrenhäuser Str. 2, 30419, Hannover, Germany; 2 Institute of Cellular and Molecular Botany, Department of Ecophysiology, University of Bonn, Kirschallee 1, 53115, Bonn, Germany; Henan Agricultural Univerisity, CHINA

## Abstract

We studied the effect of Silicon (Si) on Casparian band (CB) development, chemical composition of the exodermal CB and Si deposition across the root in the Si accumulators rice and maize and the Si non-accumulator onion. Plants were cultivated in nutrient solution with and without Si supply. The CB development was determined in stained root cross-sections. The outer part of the roots containing the exodermis was isolated after enzymatic treatment. The exodermal suberin was transesterified with MeOH/BF_3_ and the chemical composition was measured using gas chromatography-mass spectroscopy (GC-MS) and flame ionization detector (GC-FID). Laser ablation-inductively coupled plasma-mass spectroscopy (LA-ICP-MS) was used to determine the Si deposition across root cross sections. Si promoted CB formation in the roots of Si-accumulator and Si non-accumulator species. The exodermal suberin was decreased in rice and maize due to decreased amounts of aromatic suberin fractions. Si did not affect the concentration of lignin and lignin-like polymers in the outer part of rice, maize and onion roots. The highest Si depositions were found in the tissues containing CB. These data along with literature were used to suggest a mechanism how Si promotes the CB development by forming complexes with phenols.

## Introduction

Silicon (Si) as the second most abundant element in the earth crust is nearly everywhere available and is taken up by plants in its soluble form silicic acid [1]. The plant kingdom can be divided into Si accumulators, intermediate type species and Si non-accumulators according to their shoot Si concentration, which ranges from 0.1 up to 10% on a dry weight basis [2]. Si accumulating species are found among the diatoms and the horsetails (*Equisetum*), to which Si is essential, and among the grasses, including rice, maize and other cereals [3,4]. The high Si concentration in the shoot of rice and maize plants is enabled by transporters that are located in the roots and facilitate the transport of silicic acid towards the xylem [5–7]. Despite the high concentration of Si in the leaves, where it is deposited as silica, SiO_2_, Si is not considered essential for higher plants as defined by Arnon and Stout (1939) [8]. However, Si enhances plant growth and alleviates several biotic and abiotic stresses [9, 10] and therefore Si is designated a beneficial or quasi-essential element [3].

In rice plants, Si was shown to decrease the radial oxygen loss from the root to the anaerobic environment and this was accompanied by a promotion of the Casparian band (CB) formation in the exodermis and endodermis [11]. Moreover, the transcription of genes related to the synthesis of lignin and suberin, the main components of the CB, was increased by Si supply. Suberin is an apoplastic biopolymer composed of the aliphatic compounds ω-hydroxy acids, α,ω-dicarboxylic acids, fatty acids, and alcohols, as well as the aromatic phenylpropanoids p-coumaric and ferulic acid [12]. Lignin is a complex aromatic heteropolymer, which is composed mainly of the lignin monomers p-coumaryl alcohol, coniferyl alcohol and sinapyl alcohol [13, 14].

Although the promotive effect of Si on CB formation was clear, the underlying mechanism remained unclear. The fortification of the CB might be related to the availability of Si in the root tissue and if so, the Si effect should not be restricted to plants that accumulate Si in the shoot but also to Si non-accumulators. To clarify this, we investigated the effect of Si supply on the development of CB in rice and maize plants as well as in the Si non-accumulator onion (*Allium cepa*). The impact of Si on the chemical composition of the exodermal suberin was analyzed and quantified by gas chromatography-mass spectroscopy (GC-MS) and flame ionization detector (GC-FID), lignin amounts were determined photometrically. Moreover, the use of laser ablation-inductively coupled plasma-mass spectroscopy (LA-ICP-MS) enabled us to analyze the Si distribution within root cross sections of the three species. Finally, we propose a model explaining how Si could promote CB formation by interacting with chemical compounds of the CB.

## Materials and Methods

### Plant material and growth conditions

Rice (*Oryza sativa* 'Oochikara') seeds were germinated in tap water for 7 d and then placed between two layers of filter paper standing in tap water for additional 7 d. Maize (*Zea mays* 'Helix') seeds were germinated between two layers of filter paper standing in tap water for 5 d. Onion (*Allium cepa* 'Hercules I hybrid') bulbs and nug (*Guizotia abyssinica*) seeds were cultivated in peat substrate for 5 d and 14 d, respectively, and then roots were washed with tap water. Subsequently 5 plants of rice, maize, onion, nug, and *Tradescantia virginiana* were transferred to 5 L pots with nutrient solution, which was aerated for all plants except for rice plants. The composition of the nutrient solution was in mM: 1.43 NH_4_NO_3_, 0.32 NaH_2_PO_4_, 0.51 K_2_SO_4_, 1 CaCl_2_, 1.6 MgSO_4_; in μM: 1.82 MnSO_4_, 0.03 (NH_4_)_6_Mo_7_O_24_, 9 H_3_BO_3_, 0.6 ZnSO_4_, 0.15 CuSO_4_ and 35.81 Fe as sequestrene (Fe-EDDHA). In the +Si treatment, the Si concentration was adjusted to 1.07 mM (which is equivalent to 30 mg L^-1^) and Si was applied as silica gel (Roth, Karlsruhe, Germany), while the control treatment (-Si) received no Si. The pH-value was adjusted to 6.0 by addition of H_2_SO_4_ and KOH. The plants were cultivated in a growth chamber (photoperiod: 16 h light, 8 h dark; temperature 25°C day / 20°C night; relative humidity 75%; light intensity 220 μmol m^-2^ s^-1^) and nutrient solution was changed every 3 d. All plants were harvested after 21 d in nutrient solution and 2 cm root zones were sampled and stored in 70% ethanol at 4°C. Shoot and root were separated and dried at 60°C for 4 d and ground. The root growth rate was determined 1 d before harvest by marking the roots 2 cm behind the tip with a waterproof marker and measuring the growth after 24 h.

### Silicon analysis

For determination of the Si concentration in the shoot and root, 200 mg dried and ground plant matter was digested in 3 ml 65% HNO_3_, 2 ml H_2_O and 2 ml 30% H_2_O_2_ in a microwave for 12 min at 190°C and then diluted with 20 ml 10% NaOH and neutralized with HNO_3_ [15]. The Si concentration in the extract and in nutrient solution was determined photometrically at 811 nm after addition of 250 μl dye reagent (0.08 M sulphuric acid and 2% ammonium heptamolybdate), 250 μl 3.3% tartaric acid and 250 μl 0.4% ascorbic acid to 50 μl of samples [16].

### Histochemical examination of roots

From each of the 4 replicates, 5 roots without lateral roots were taken for cross sectioning and from 5 cross sections 20 cells each were used for microscopic examination, so the degree of development of CB was calculated on basis of 400 cell walls per treatment. The root zones, where CB initiated under-Si conditions (zone A), and older root zones 4 cm behind (zone B) were harvested. Root zone A was at 0–2 cm distance from the root tip (drt) (rice, onion), 2–4 cm drt (maize), 6–8 cm drt (*Tradescantia*), and at 8–10 cm drt (nug), root zone B was at 4–6 cm drt (rice, onion), 6–8 cm drt (maize), and at 12–14 cm drt (nug). About 0.5 cm from both edges of the 2 cm-zone were removed and free hand cross sections were made from the remaining middle part of the root zone.

For detection of CB, free hand cross sections of plant roots were stained with 0.1% (w/v) berberine hemi-sulphate for 60 min and with 0.5% (w/v) aniline blue for further 30 min [17]. Stained sections were mounted in 0.1% (w/v) FeCl_3_ in 50% (v/v) glycerine and examined using an Axioskop fluorescence microscope (Zeiss, Jena, Germany) with UV illumination and excitation filter G 365, chromatic beam splitter FT 395 and barrier filter LP 420. Pictures were taken with the AxioCam MRc (Zeiss) and picture recording software (AxioVision Ac, Version 4.4, Zeiss). Under UV light, suberin exhibited a blue-white colour. The development of CB in the anticlinal exodermal cell walls was determined and allocated to one of four stages: 0% (stage I), 0–25% (II), 25–50% (III) and 50–100% (IV) development of CB in the anticlinal cell wall of the exodermis.

For comparative visualization of CB and suberin lamellae (S5 Fig), roots of onion, rice and maize were infiltrated with fixation solution containing 3.7% (v/v) formaldehyde, 3.7% (v/v) glutaraldehyde in PBS buffer (137 mM NaCl, 2.7 mM KCL, 10 mM Na_2_HPO_4,_ 1.8 mM KH_2_PO_4_) for 12 h at 8°C in glass vials. After fixation roots were dehydrated using successive baths of 70, 80, 90 and 100% of ethanol for 1 h each on a radial shaker. Then, dehydrated roots were incubated for 1 h at 39°C in a 1:1 mixture of ethanol and steedman´s wax (9:1 Polyethylenglycol: 1-Hexadecanol). For embedding roots were infiltrated with 100% steedman´s wax overnight, gently shaking at 39°C. On the next day the wax was changed 2 times with 1 h incubation. Fixed roots were then placed vertically into a holder and chilled at RT overnight. From the imbedded roots, 10 μm serial sections were cut using a microtome (Hyrax M55, Zeiss, Jena, Germany) placed on glass slides (SuperFrost®, Carl Roth, Karlsruhe) on a drop of water to unfold on 32°C (Medox Type 14801 heating plate). Afterwards, water was removed and slides were dried overnight followed by de-waxing on 40°C using 100% pre-warmed ethanol. Staining was done directly on the glass slides. The berberine-aniline blue-staining was applied as described above with the exception of 3 x 5 min each washing steps after berberine hemi-sulphate staining and 3 x 10 min each washing steps after 0.5% aniline blue staining. For fluorol yellow 088 staining a modified protocol from Brundrett, Enstone & Peterson (1988) [17] and Lux et al. (2005) [18] was applied. A fresh 0.001% solution of fluorol yellow 088 was prepared in lactic acid, then heated to 70°C and filtrated using a 20 μm filter.

Pictures were taken with the AxioCam MRc (Zeiss) and picture recording software (AxioVision Ac, Version 4.4, Zeiss). Under UV light, suberin exhibited a blue-white colour. The development of CB in the anticlinal exodermal cell walls was determined and allocated to one of four stages: 0% (stage I), 0–25% (II), 25–50% (III) and 50–100% (IV) development of CB in the anticlinal cell wall of the exodermis.

Microscopy of berberine-aniline blue staining of CB was done using a Axioplan I fluorescence microscope (Zeiss, Jena, Germany) and picture recording software (AxioVision Ac, Version 4.8, Zeiss). Fluorol Yellow 088 staining was visualized using a GFP filter with an excitation filter BP 485, chromatic beam splitter FT 510 and barrier filter LP 520. Suberin should appear in a bright yellow / green fluorescence signal. Pictures were adjusted in brightness using the ImageJ-software 1.50a, additionally fluorescence pictures of onion were equally enhanced by 0.1% true colour contrast.

### Cell wall isolation and preparation for suberin and lignin analysis

The root surface of harvested root zones was scanned using WinRHIZO software (Regent Instruments Inc., Quebec, Canada) in order to relate components of the outer part of the root to root surface. The cell wall isolation and preparation was performed as described in detail previously [19]. Briefly, root zone B of rice, maize and onion plants were washed with distilled water and then incubated at room temperature for 4 d in 1 ml enzyme solution (0.1 M citric acid monohydrate, 1% pectinase (v/v), 1% cellulase (v/v), 0,1% NaN_3_), which was renewed daily. After enzymatic digestion the non-degradable outer part of the root comprising the exodermal cell wall fraction was separated from the tissue containing the stele by use of two forceps under a binocular. The exodermal cell wall material was incubated in enzyme solution for another 2 d to remove any residual cortex material. Afterwards the isolates were washed with distilled water and incubated in borate buffer (0.01 M sodium borate, pH 9) for 2 d.

Isolated cell wall material, dried for 1 d at 60°C, was extracted for 5 d with a 1:1 mixture of chloroform and methanol, which was changed daily. After the extraction the isolated samples were dried for 2 h in the desiccator over silica gel. The dry weight was determined directly before suberin and lignin analysis of the isolated samples.

### Suberin analysis

For transesterification, the dried isolates were incubated for 16 h in 1 N methanolic boron trifluoride (MeOH/BF_3_; Fluka) at 70°C. Saturated NaCl was added to stop the transesterification reaction and to advance the following phase separation. Dotriacontan (C_32_ alkane, 10.025 mg / 50 ml) was added as internal standard to each sample. The soluble hydrophobic components were extracted by adding chloroform. The chloroform phase was transferred to a new vial and extraction was repeated three times. The extract was dried with water free Na_2_SO_4_ and the volume was reduced to 50 μl by evaporation under N_2_ flow.

Samples were derivatized in 20 μl BSTFA (N,N-bis(trimethylsilyl)-trifluoracetamide; Machery-Nagel, Düren, Germany) and 20 μl dry pyridine (GC-grade, Merck, Darmstadt, Germany) for 40 min at 70°C. Pyridine catalyzed the derivatization reaction and BSTFA masked free hydroxyl- and carboxylgroups forming the corresponding trimethylsilyl derivatives [20]. Samples were analyzed by gas chromatography (GC; Type: 6890N, Agilent Technologies, Santa Clara, USA) and mass spectroscopy (MS; Type: 5973N, Agilent Technologies). The GC and MS analysis was performed as described in detail previously [21]. The quantification of the monomers was performed using a gas chromatograph combined with a flame ionization detector. Four replicates of each plant species were used.

### Lignin analysis

0.5 mg of isolated and extracted cell wall material was solubilized in 1 ml acetyl bromide/acetic acid (1:3, v/v) in a loosely capped glass tube for 30 min at 70°C. Afterwards the tube was cooled down to 15°C and 0.9 ml NaOH and 5 ml glacial acetic acid were added to hydrolyze excess acetyl bromide. Bromine (Br) and polybromide were destroyed by adding 0.1 ml hydroxylamine-HCl. The solution was diluted to a total volume of 10 ml with acetic acid. Within 10–15 min after cooling down the absorption was read at 280 nm with a fused glass microplate (9 mm layer thickness) on a microplate reader (μQuant, BioTek Instrument, Inc., Winooski, USA). The resulting absorbance was multiplied by 1.11 to correct for a cuvette with 10 mm thickness, where a lignin concentration of 14 μg ml^-1^ led to an absorption of 0.343 [22]. Four replicates of each plant species were used.

### Embedding and sectioning of roots for LA-ICP-MS

Root zone B of rice, maize and onion plants grown in +Si nutrient solution were embedded using the Steedman’s wax protocol [23] in a modified form. Roots were fixed in freshly prepared Farmer’s fixative (3 parts ethanol + 1 part acetic acid) at 4°C overnight. Roots were then dehydrated under rotation for each 2 h in 75%, 85%, 95% and 100% ethanol, respectively. Molten Steedman’s wax (9 parts poly (ethylene glycol) distearate (SigmaAldrich, St. Louis, USA) + 1 part 1-hexadecanol (SigmaAldrich)) was mixed 1:1 with ethanol and roots were incubated in the mixture at 38°C overnight. Roots were then incubated three times at 38°C for 2 h each in pure Steedman’s wax. Afterwards, roots were divided in 3 mm pieces and embedded in Steedman’s wax in TurbOflowII molds and cassettes (McCormick Scientific, St. Louis, USA). The wax was allowed to solidify overnight at room temperature. The wax blocks were cut with a Hyrax M55 rotary microtome (Zeiss, Jena, Germany) into slices of 20, 50 and 100 μm. Wax slices were dissolved by addition of ethanol and root sections were washed several times by exchanging ethanol until complete removal of the wax.

### Laser ablation-inductively coupled plasma-mass spectroscopy

Root sections floating in ethanol were transferred to Tin (Sn) foils (Elementar Analysensysteme GmbH, Hanau, Germany) placed on microscopy glass slides. Evaporation of the ethanol fixed the root sections to the foil, which allowed the use of a laser for ablation. For rice and maize, 100 μm thick root sections were used and for onion, 50 μm thick slices were used. Root tissue was ablated with the solid state NYAG-laser UP193 SS (New Wave Research Co. Ltd., Cambridge, England). The laser beam was adjusted to a diameter of 75 μm and energy of 2.5 J cm^-2^ for rice and maize and to a diameter of 50 μm and energy of 4.0 J cm^-2^ for onion. The pulse length was 1 s and the frequency was 10 Hz. The ablation chamber was coupled to the ICP-MS torch with a tygone^®^ tube and was filled with carrier gas at a flow rate of 0.25 L min^-1^. After the chamber was passed the flow rate was increased with makeup gas to 1.2 L min^-1^. ^13^C and ^28^Si signals were detected using the quadrupole ICP-MS 7500 CX (Agilent Technologies, Santa Clara, USA). The Si abundance in the root tissue was calculated as ^28^Si:^13^C ratio and expressed in relation to the maximum value, which was set to 1.0.

### Statistical analysis

All treatments were replicated four times and mean of the treatments were compared with t-test using R software [24]. For comparison of the developmental stages of CB, a cumulative link mixed model was calculated with p < 0.05 using the package ordinal in R software.

## Results

### Plant growth, Si concentrations and Casparian bands

**Fig 1:**
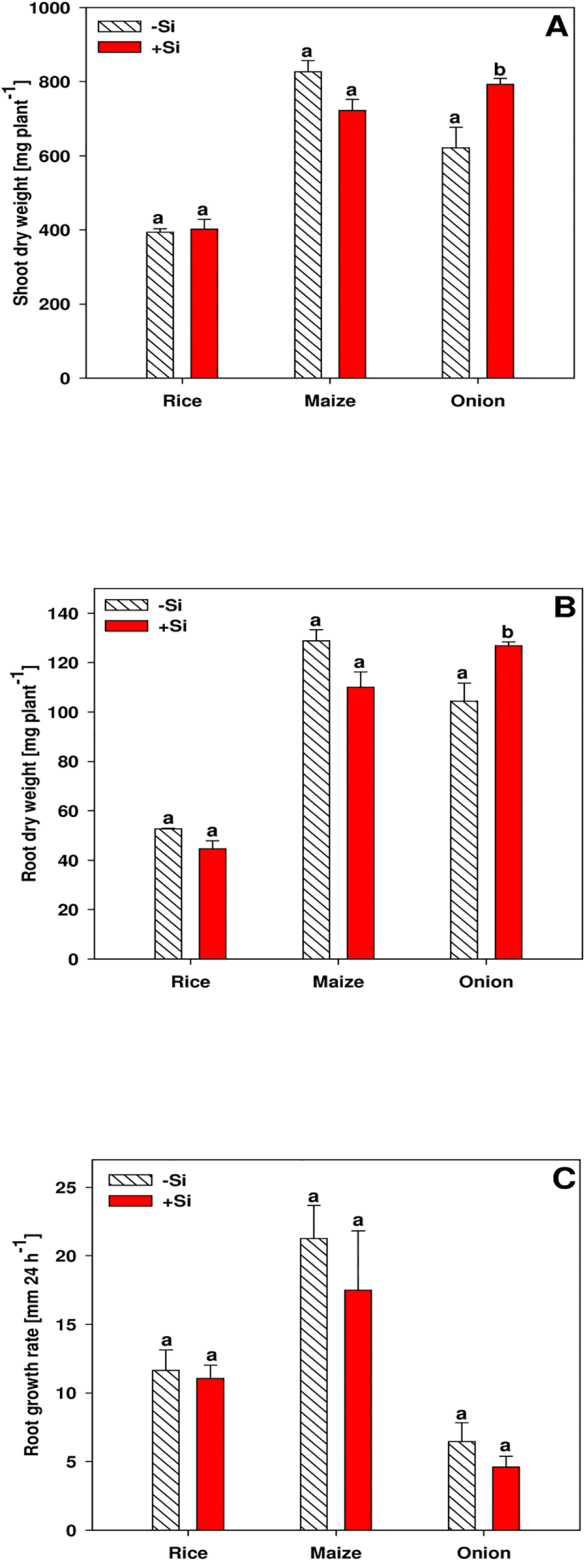
Shoot (A) and root (B) dry weight and root growth rate (C) of rice, maize and onion plants as affected by Si supply. Data are mean ± s.e., n = 4. Different letters indicate a significant difference between Si treatments of a species; t-test with p < 0.05.

The shoot dry weight of rice and maize plants was not affected by Si supply, while Si supply increased the shoot dry weight of onion plants (Fig 1A). Similarly, the root dry weight of onion plants was slightly higher in +Si than in-Si plants, while the root dry weight of rice and maize plants did not differ between the Si treatments (Fig 1B). The root growth rate was highest for maize and lowest for onion, however, root growth of no plant species was affected by Si supply (Fig 1C).

**Fig 2:**
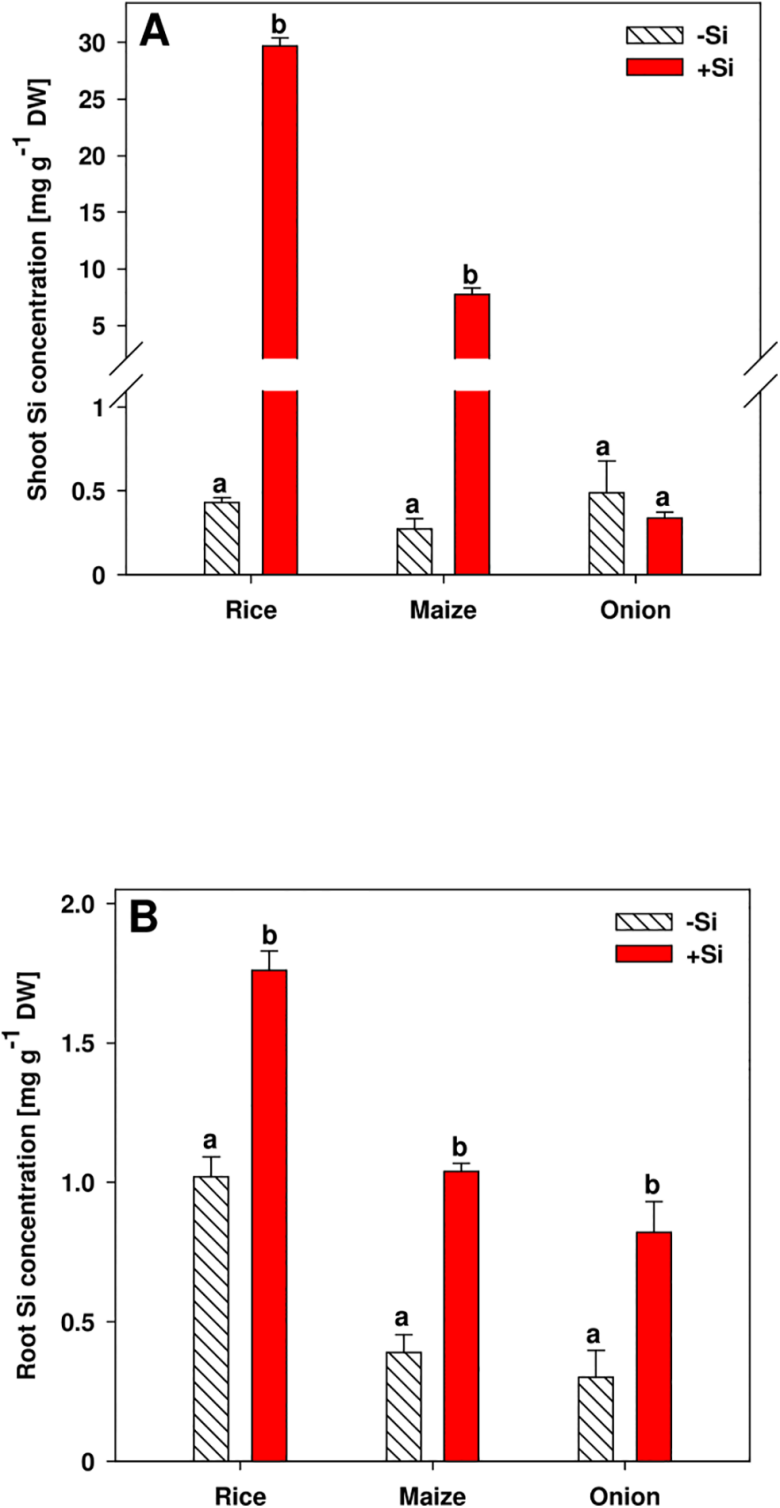
Silicon concentration in shoot (A) and root (B) of rice, maize and onion plants as affected by Si supply. Data are mean ± s.e., n = 4. Different letters indicate a significant difference between Si treatments of a species; t-test with p < 0.05.

The Si concentration in the shoot of rice and maize plants was clearly increased when Si was supplied, while in onion shoots the Si concentration did not differ between the Si treatments (Fig 2A). In contrast, all species accumulated additional Si in the roots when Si was supplied (Fig 2B).

**Fig 3:**
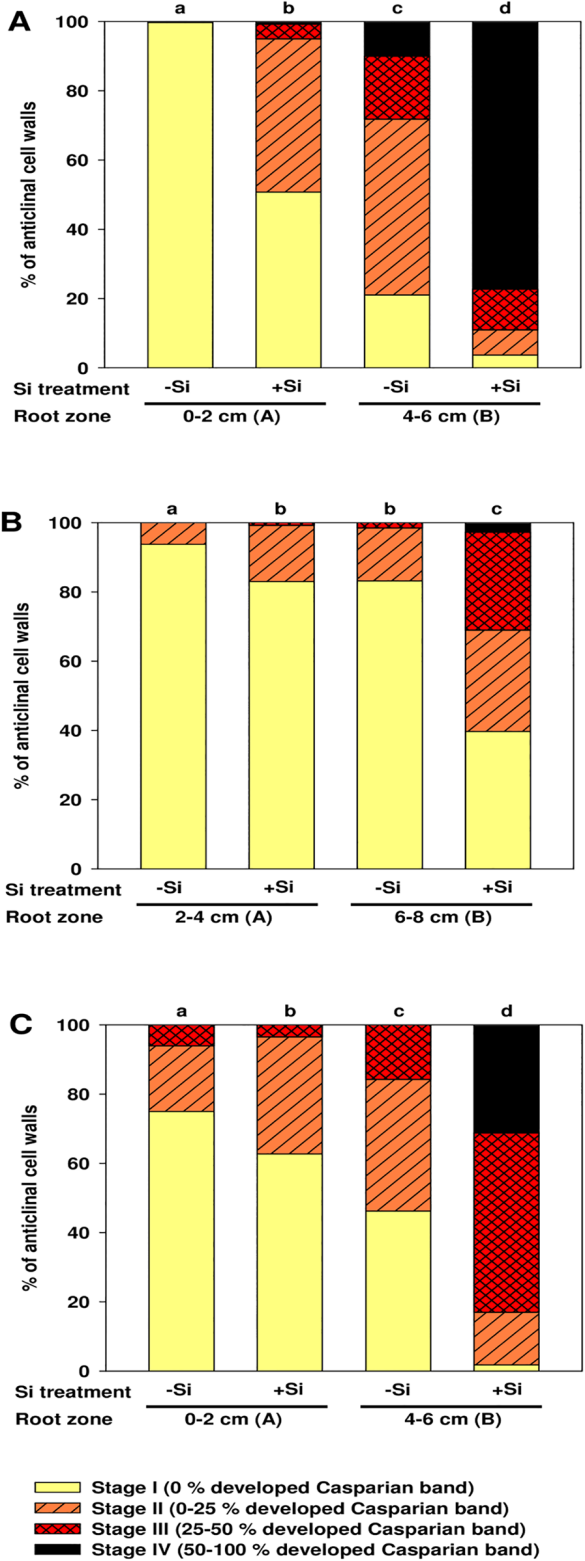
Development of Casparian bands (CB) in the exodermis of rice (A), maize (B) and onion (C) roots as affected by Si supply. Root zone where the formation of CB initiated was defined as root zone A, which was at 0–2 cm drt for rice and onion, and at 2–4 cm drt for maize. Root zone B started 2 cm behind root zone A. Exodermal CB were classified into stages I-IV according to 0, 0–25, 25–50 or > 50% of the length of the anticlinal cell wall with developed CB. n = 4. Different letters indicate significant difference between Si treatments and root sections of a species; cumulative link mixed models with p < 0.05.

The formation of exodermal CB started in different distances from the root tip (zone A). In rice and onion roots the CB was already developed at 0–2 cm drt, while in maize roots CB initiated at 2–4 cm drt and even later in *Tradescantia* (6–8 cm drt) and nug (8–10 cm drt) (Fig 3; S3 and S4 Figs). Si supply promoted the formation of exodermal CB in all plant species in both, young (zone A) and older root parts (zone B: 4–6 cm drt for rice and onion; 6–8 cm drt for maize; 12–14 cm drt for nug). The development of suberin lamellae was similar to the development of CB in rice, maize and onion exodermis (S5 Fig).

### Suberin and lignin

**Fig 4:**
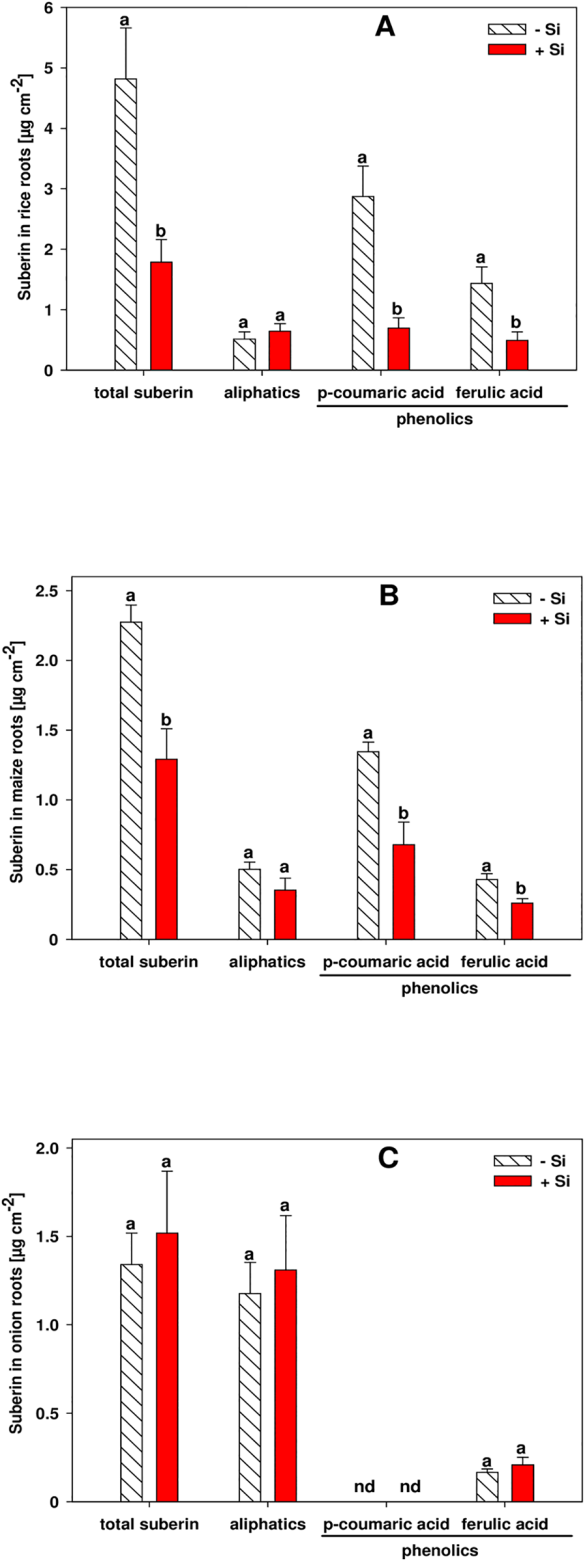
Total, aliphatic and phenolic suberin amounts in the outer cell layers comprising the exodermis of root zone B in rice (A), maize (B) and onion (C) as affected by Si supply. Amounts were determined via GC-FID. Root zone B was at 4–6 cm drt in rice and onion and at 6–8 cm drt in maize roots. Data are mean ± s.e., n = 4. Different letters indicate a significant difference between Si treatments of a suberin fraction; *t*-test with p < 0.05. nd = not detected.

The composition and quantity of suberin was determined in the outer cell layers comprising the exodermis in the root zone B of rice, maize and onion roots. In rice and maize roots the total suberin was decreased by Si supply. This was due to reduced amounts of the phenolic compounds p-coumaric acid and ferulic acid (Fig 4A and 4B). The sum of the aliphatic suberin monomers was not decreased by Si supply, although single aliphatic compounds were affected by Si supply in rice and maize (S1 Fig). In contrast, the Si treatments did not affect any suberin fraction in onion roots (Fig 4C). Moreover, ferulic acid was the only phenolic suberin compound in the onion root, while p-coumaric acid was not detected.

**Fig 5:**
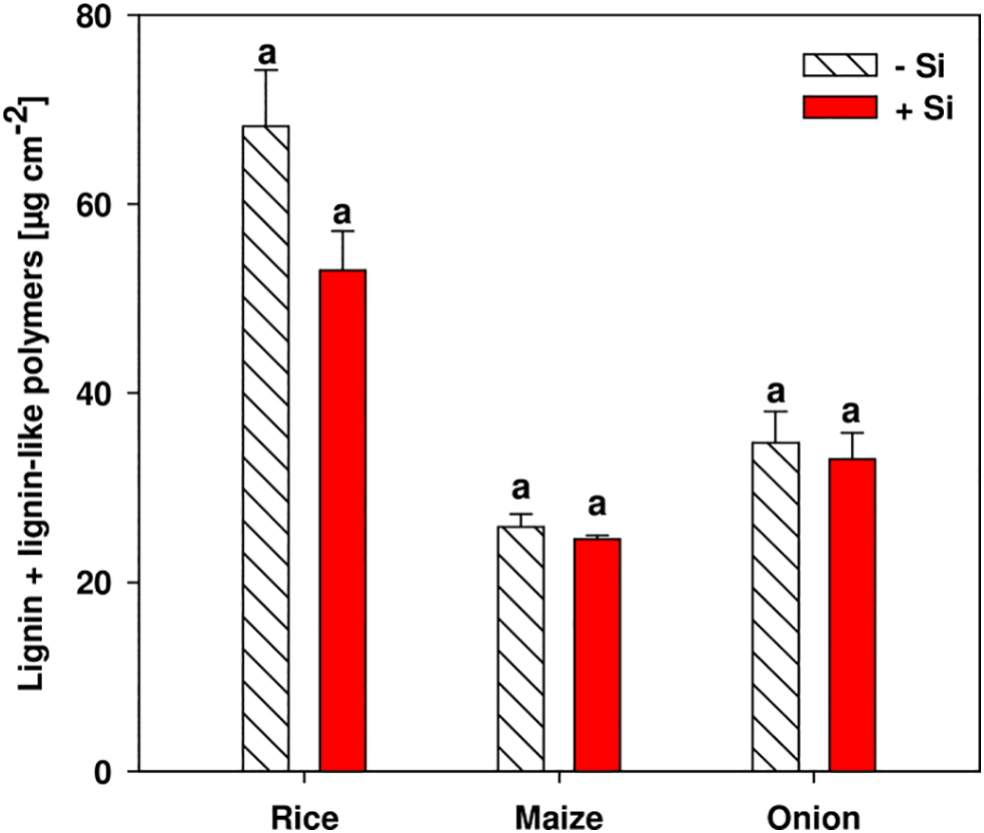
Concentration of lignin and lignin-like polymers in the outer cell layers of the root comprising the exodermis and the sclerenchyma in rice, and the exodermis in maize and onion as affected by Si supply. Amounts were determined photometrically. Root zone B was at 4–6 cm drt in rice and onion and at 6–8 cm drt in maize roots. Data are mean ± s.e., n = 4. Different letters indicate a significant difference between Si treatments of a plant species; *t*-test with p < 0.05.

The content of lignin and lignin-like polymers in the outer cell layers of the root comprising the exodermis and the sclerenchyma in rice, and the exodermis in maize and onion was not affected significantly by Si supply (Fig 5). The lignin concentration in rice was about two times higher than in maize and onion roots.

### Distribution of Si in the root

**Fig 6:**
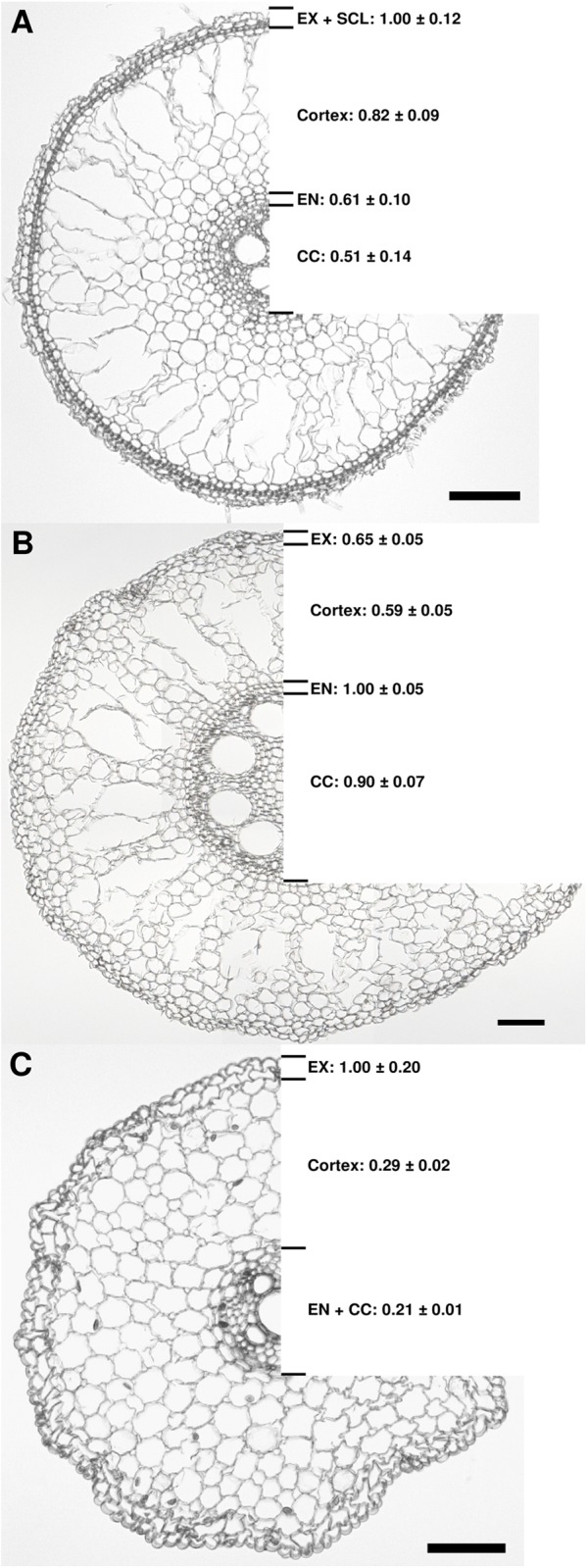
Distribution of Si in the root zone B of rice (A), maize (B) and onion (C). The values indicate the relative Si abundance relating to a maximum value of 1.0. Root zone B was at 4–6 cm drt in rice and onion and at 6–8 cm drt in maize roots. Data are mean ± s.e., n = 4. The black bar indicates 100 μm. EX, exodermis; SCL, sclerenchyma; EN, endodermis; CC, central cylinder. The formation of an aerenchyma was observed not only in rice but also in maize roots, which was reported before for well-aerated maize plants [61].

The Si distribution in root zone B of rice, maize and onion roots was determined by analyzing the ^28^Si:^13^C ratio using LA-ICP-MS. The Si abundance was expressed in relation to the maximum value which was set to 1.0. In the rice root, the Si abundance was highest in the outer cell layers comprising the exodermis and the sclerenchyma and gradually decreased towards the central cylinder (Fig 6A). In the maize root, the Si signal was highest in the endodermis, while the Si signal was only slightly higher in the exodermis than in the cortex (Fig 6B). The highest Si concentration in the onion root was found in the outer region comprising the exodermis, while it was clearly lower in the cortex as well as endodermis and central cylinder (Fig 6C).

## Discussion

### Plant growth, Si concentration and Si distribution

Silicon supply did not affect the growth of rice and maize plants, while onion shoot and root dry weight was increased when grown in +Si nutrient solution (Fig 1A and 1B). It is well-known that Si may enhance the growth of rice plants when cultivated in rice paddy fields [3, 25, 26]. However, a growth promotion of rice plants by Si supply was not observed in other studies with rice cultivated in nutrient solution [11]. The reason might be the optimal cultivation conditions in nutrient solution in a climate chamber where no stress could be alleviated by Si application [3]. By contrast, a growth promotion of onion plants is in contradiction to other reports in literature [25]. A higher initial dry matter could be the reason for higher final dry matter yield in the +Si treatment, since the onion bulbs were inhomogeneous. This is further supported by the fact that Si did not affect root growth rates in onion and in the other two plant species (Fig 1C), which is in line with previous data [11].

The Si concentrations in the shoots of rice, maize and onion plants grown in-Si nutrient solution were on a similar level, while shoot Si concentrations were clearly enhanced in rice and maize plants when Si was supplied (Fig 2A). By contrast, onion did not accumulate additional Si in the shoot when grown in +Si nutrient solution. This is in line with literature that classified onion as Si non-accumulator [25]. Moreover, rice has a high ranking in the extensive list of Hodson et al. [4], who ranked 735 plant species according to their shoot Si concentration, followed by maize, while the onion family *Amaryllidaceae* has a very low ranking. The Si concentrations in the root, however, were enhanced by Si supply in all species (Fig 2B).

The concentration of Si in the rice roots was generally highest in the outer part of the root comprising the exodermis and the sclerenchyma (Fig 6A). Silica depositions in the rice root were found in the tangential and in the radial cell walls of the endodermis [27], while Si accumulation in other studies was reported not only in the endodermis but also in the exodermis of rice roots [28, 29]. More recently, Moore et al. [30] confirmed silica deposition in the cell walls of both, endodermis and exodermis, as well as in the sclerenchyma.

The highest Si concentration in the maize root was found in the endodermis, while in onion roots, the exodermis contained the most Si (Fig 6B and 6C). Differently from rice, the Si distribution in maize and onion roots had not been reported before. However, Si accumulation in the endodermis has also been reported for other grasses, including sorghum, barley, oats, and wheat [31, 32].

### Casparian bands

Exodermal CB in rice and onion roots was already initiated in the root tip (0–2 cm), while CB in the maize root exodermis were detected in the root zone at 4–6 cm (Fig 3). This is in line with literature, where the development of exodermal CB was reported at 3 cm drt in rice plants grown in nutrient solution [33] or even at 1 cm drt when grown in stagnant deoxygenated medium [34, 35]. Comparative analysis made by Schreiber et al. [20] revealed that the exodermis was already fully developed at 6 cm drt in rice roots, but was not yet complete at 12 cm in maize roots. Onion plants grown either in vermiculite or in nutrient solution showed CB in the exodermis at 5 cm drt [36, 37].

Generally, the formation of exodermal CB at a short distance from the root tip coincided with a low root growth rate in onion and rice compared to maize (Fig 1C), which is in accordance with studies reporting an earlier development of CB in rice and maize exodermis when root growth was reduced [29, 38, 39]. The formation of CB was enhanced in all plant species when Si was supplied (Fig 3). This confirms previous observations in rice [11], while it was reported for the first time for maize and onion. Si also induced suberin lamaellae formation (S5 Fig). The simultaneous development of CB and suberin lamaellae in the exodermis agrees with literature [47]. This Si effect did not result from slowed root growth, since root growth rates were not different between Si treatments.

Similar Si effects on CB formation in the exodermis were also observed in the *commelinoid* monocotyledon *Tradescantia virginiana* and in the dicotyledon *Guizotia abyssinica* (S3 and S4 Figs). It was shown in a previous study that the Si-induced CB formation reduced the radial oxygen loss of the rice root [11], thus providing a physiological proof of the Si impact. It was observed in other studies that Si reduced the translocation of sodium and chloride from root to shoot in rice plants, and this was correlated with a reduced apoplastic bypass flow along the root [29, 40, 41]. The latter was suggested to be the result of silica deposition at the endodermis or exodermis. However, the limited bypass flow can also be explained by an enhanced formation of exodermal CB after Si treatment.

Besides the earlier induction of CB formation by Si in roots of all species, the accumulation of Si in the different tissues correlates with the occurrence of exodermal apoplastic barriers. In rice and onion, where the apoplastic pathway for water and solutes is limited by the early formation of exodermal CB in root zone B (Fig 3A and 3C), Si accumulates at this border tissue (Fig 6A and 6C), whereas in maize, where CB develop much later in the exodermis (Fig 3B), the highest Si concentration is located at the endodermis (Fig 6B). This accumulation probably increases the promotional effect on CB formation.

### Suberin and lignin

A promoted formation of CB would suggest increased amounts of its potential components suberin and lignin, but surprisingly this was not found. By contrast, Si did not affect the suberin in the outer part of the onion root and even decreased the total suberin content in the outer cell layers of rice and maize root (Fig 4A, 4B and 4C). This decrease was due to a reduced quantity of the aromatic suberin, while the aliphatic suberin fraction in all plants was unaffected by Si supply. Si did not affect suberin in onion roots probably because the phenolic suberin fraction was only 13% of the total suberin in onion, while it was 76% and 83% in maize and rice, respectively. Moreover, the main components of the phenolic suberin in rice and maize roots were ferulic acid and p-coumaric acid, which is in line with literature [20, 34, 35, 42]. By contrast, the phenolic suberin in the outer part of the onion roots consisted only of ferulic acid, and lacked p-coumaric acid, which is in accordance with a previous study [43]. The amounts of the phenolic and aliphatic suberin in the outer part of the rice and maize root were in the same order of magnitude as reported in literature [20, 34, 35] and the amount of aliphatic suberin in the outer part of the onion root was also in similar values to the literature [44]. The formation of exodermal CB along the maturing onion root is accompanied by an increase of the insoluble aliphatic suberin [43]. In our study, this positive correlation between CB development and aliphatic suberin was not found. However, we observed microscopically a simultaneous development of CB and suberin lamaellae (S5). It is assumed that the fluorescent dye fluorol yellow is a much more sensitive indicator of suberin lamellae initiation than the chemical analysis.

The composition of the aliphatic suberin fraction was different between rice, maize and onion roots and was less complex in onion than in rice and maize (S1 Fig). The group of ω-hydroxy fatty acids was the most abundant substance class of the aliphatic suberin in the outer part of rice, maize and onion roots, which is congruent with previous studies [20, 35, 42, 44]. The lignin and lignin-like polymer concentration in the outer part of the root was not affected by the Si treatments in any plants (Fig 5). The concentration in rice was twice as high as in maize and onion, probably as a result of the phenol-rich sclerenchyma. The concentration of lignin when expressed on a dry weight basis (S2 Fig) was in the range reported for onion, maize and rice roots [34, 35, 44, 45].

The composition of CB is controversially discussed in literature and it is thought that suberin and lignin are the main components [46, 47]. By contrast, Naseer et al. [48] concluded from studies with *Arabidopsis thaliana* that lignin is indispensable for fully functional CB while suberin is not. On the other hand, lignin was found along with suberin in endodermal CB of several other plants including monocotyledonous and dicotyledonous species [21, 49, 50]. However, Geldner [51] argued that the suberin found is not essential for the formation or part of the CB but rather deposited as suberin lamellae during the second stage of endodermal differentiation.

Independent of the question whether suberin is an integral part of the CB, it was surprising to see that Si, on the one hand, promoted the formation of CB in rice, maize and onion exodermis, but, on the other hand, did not affect or rather decreased the amount of suberin, while the total phenol or lignin concentration was not affected. How can this paradox be explained?

### A model of Si and phenol interaction

The Si-enhanced formation of CB in the exodermis was proven both by microscopical and physiological results and data of the suberin and lignin analysis need to be interpreted. Prior to the suberin analysis, the suberin polymer is depolymerized to its monomers in a transesterification reaction with MeOH/BF_3_ [44, 52]. This reaction converts acids that are esterified in a polymer into their respective methylesters [21]. However, monomers connected by bonds other than ester bonds are not cleaved by this method and thus, these monomers are not released and cannot be detected by GC analysis. On the other hand, the lignin analysis is based on a depolymerization reaction using acetyl bromide/acetic acid, which is much more aggressive than the MeOH/BF_3_ method and allows complete dissolution of lignin [22]. Other phenolic depolymerization methods resulting in complete dissolution of lignin and lignin-like polymers, such as nitrobenzene oxidation [53] or cupric oxide oxidation [54], would not allow deeper insights since Si did not affect the concentration of lignin and lignin-like polymers, as determined by the acetyl bromide/acetic acid assay. If Si-complexed phenols form non-ester bonds, the amount of detectable aromatic suberin monomers, using the MeOH/BF_3_ assay, would be decreased by Si, while the amount of lignin as detected by the acetyl bromide/acetic acid assay would not be affected.

An increase of the lignin and lignin-like polymers due to Si supply could not be observed although the histochemical examination of exodermal CB indicated an increase of this fraction since the dye berberine-aniline blue has a major affinity for phenolic compounds [17]. This effect revealed by microscopy was presumably not observed in the analytic results since the phenolics contained in the stained fraction were a magnitude lower than total lignin and lignin-like polymers in the same root section (Figs 4 and 5).

An interaction of Si and phenols was discussed several times in literature. It was reported in the early-1970s that silica gel or colloidal silica can form complexes with catechol as six-coordinated Si [55, 56]. Inanaga and Okasaka [57] proposed that Si was associated with phenols in root cell walls of rice and could lead to crosslinking between lignin and carbohydrates. Support for this hypothesis was yielded from *in vitro* studies, where lignin isolated from rice induced the deposition of silica [58]. Moreover, callose was reported to induce silica deposition [59], which is in line with data from the Si-accumulator horsetail (*Equisetum arvense*), where Si was found along with callose in the cell walls [60].

Considering the chemical reactivity of Si with phenols, the accumulation of Si in the exodermis and the promotion of the CB in the exodermis, we hypothesize that Si either crosslinks phenols with the cell wall or induces precipitation of the phenols leading to an enhanced development of CB. This hypothesis considers that Si accumulated in phenol-rich compartments, such as the exodermis and endodermis, and that Si reduced the amount of esterified phenols as determined after depolymerization using the MeOH/BF_3_ method whereas the total phenol (esterified and non-esterified complexed phenols) concentration as detected with the lignin determination method was not affected.

In conclusion, we demonstrated that Si enhanced the formation of exodermal CB not only in roots of rice, but also in maize and onion plants as well as in nug and *Tradescantia*. This promotion was not due to increased suberin amounts in the outer part of the roots. Instead, we observed a decrease of esterified phenolic compounds in rice and maize and we suggest that Si induced enhancement of CB might be the result of a chemical interaction of phenolic compounds with Si. The data presented and literature reports provide solid ground for the hypothesized Si mechanism enhancing CB in the exodermis.

## Supporting Information

S1 Fig Amounts of aliphatic suberin compounds in the outer cell layers of rice, maize and onion roots. (PDF)

S2 Fig Amount of the lignin and lignin-like polymers in the outer cell layers of rice, maize and onion roots. (PDF)

S3 Fig Development of Casparian bands (CB) in the exodermis of nug plants as affected by Si supply. (PDF)

S4 Fig Development of Casparian bands (CB) in the exodermis of *Tradescantia virginiana* plants as affected by Si supply. (PDF)

S5 Fig Formation of Casparian bands and suberin lamellae in the exodermis of rice, maize and onion roots as affected by Si supply. (PDF)
