# Supplementary material for: Silicon Promotes Exodermal Casparian Band Formation in Si-Accumulating and Si-Excluding Species by Forming Phenol Complexes
Source: PLoS One. 2015 Sep 18;10(9):e0138555. doi: 10.1371/journal.pone.0138555 (PMC4575055; doi:10.1371/journal.pone.0138555)

Figure S1. Amounts of aliphatic suberin compounds in the outer cell layers of rice (A), maize (B) and onion (C) roots.

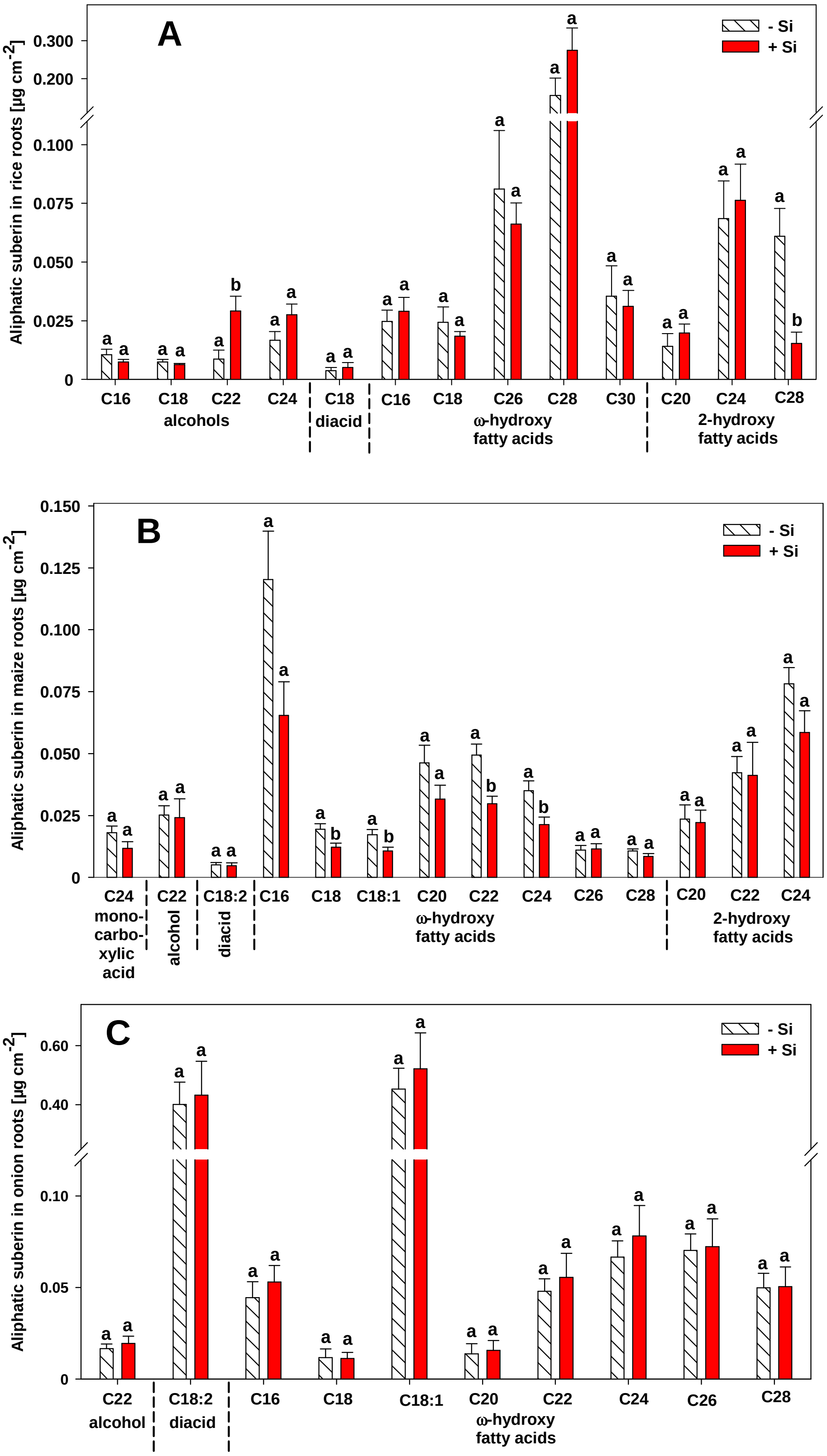

Supplement: S1 Fig — (PDF) [file pone.0138555.s001.pdf]
