# Supplementary material for: Silicon Promotes Exodermal Casparian Band Formation in Si-Accumulating and Si-Excluding Species by Forming Phenol Complexes
Source: PLoS One. 2015 Sep 18;10(9):e0138555. doi: 10.1371/journal.pone.0138555 (PMC4575055; doi:10.1371/journal.pone.0138555)
Supplement: S5 Fig — (PDF) [file pone.0138555.s005.pdf]

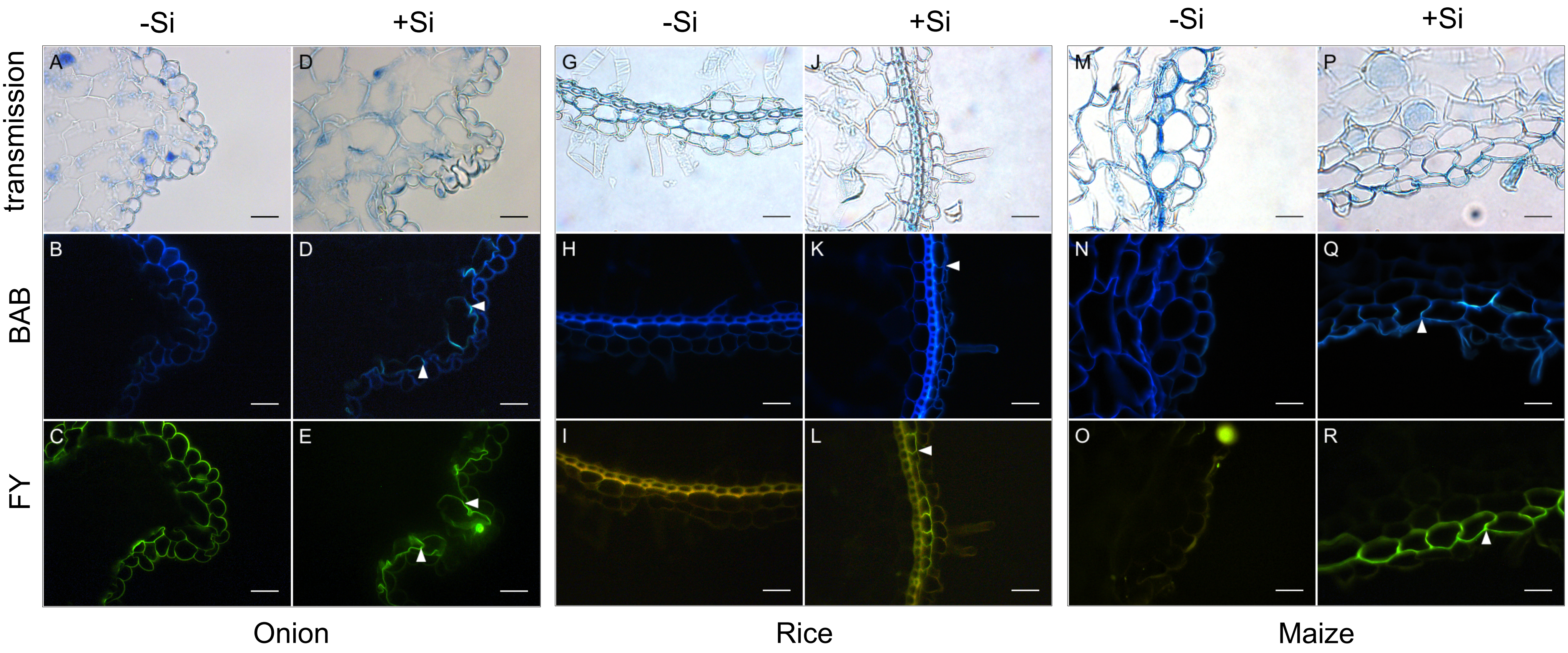

Figure S5: **Formation of Casparian bands and suberin lamellae stained with berberine-aniline blue and fluorol yellow, respectively, in the exodermis of rice, maize and onion roots as affected by Si supply**. Serial sections of the same root were stained with berberine-aniline blue (BAB: B,E,H,K,N,Q) or fluorol yellow 077 (FY: C,F,I,L,O,R), the range of 4 cm behind the root tip is shown for onion (A-F) and 6 cm for rice (G-L) and maize (M-R). Arrows indicate formation of CB or suberin lamellae. Bar: 25 µm.
